# The Cellular p53 Inhibitor MDM2 and the Growth Factor Receptor FLT3 as Biomarkers for Treatment Responses to the MDM2-Inhibitor Idasanutlin and the MEK1 Inhibitor Cobimetinib in Acute Myeloid Leukemia

**DOI:** 10.3390/cancers10060170

**Published:** 2018-05-31

**Authors:** Katja Seipel, Miguel A. T. Marques, Corinne Sidler, Beatrice U. Mueller, Thomas Pabst

**Affiliations:** 1 Department for Biomedical Research (DBMR), University of Bern, 3008 Bern, Switzerland; Miguel.Teixeiramarques@students.unibe.ch (M.A.T.M.); Corinne.Sidler@dbmr.unibe.ch (C.S.); 2 Department of Medical Oncology, University Hospital Bern, 3010 Bern, Switzerland; Beatrice.Mueller@insel.ch

**Keywords:** acute myeloid leukemia (AML), FMS like tyrosine kinase 3 (FLT3), tumor suppressor p53 (TP53), mouse double minute 2 homolog (MDM2), mitogen-activated protein kinase kinase (MEK; MAP2K; MAPKK)

## Abstract

The tumor suppressor protein p53 is inactivated in a large variety of cancer cells. Cellular p53 inhibitors like the mouse double minute 2 homolog (MDM2) commonly suppress the p53 function in acute myeloid leukemia (AML). Moreover, fms like tyrosine kinase 3 (FLT3) growth factor signaling pathways including the mitogen-activated kinase (MAPK) cascade (RAS-RAF-MEK-ERK) are highly active in AML cells. Consequently, the combined administration of MDM2 and MEK inhibitors may present a promising anti-leukemic treatment strategy. Here we assessed the MDM2 antagonist idasanutlin and the MEK1 inhibitor cobimetinib as single agents and in combination in a variety of AML cell lines and primary AML blast cells for their ability to induce apoptosis and cell death. AML cell lines and blast cells comprised all major AML subtypes based on the mutational status of *TP53, FLT3* and *NPM1* genes. We observed a considerably varying anti-leukemic efficacy of idasanutlin and cobimetinib. AML cells with high sensitivity to the single compounds as well as to the combined treatment emerged with normal karyotype, wild-type *TP53* and elevated FLT3 and MDM2 protein levels. Our data indicate that AML cells with normal karyotype (NK) and wild-type status of *TP53* with elevated FLT3 and MDM2 expression emerge to be most sensitive to the combined treatment with cobimetinib and idasanutlin. FLT3 and MDM2 are biomarkers for treatment response to idasanutlin and cobimetinib in AML.

## 1. Introduction

Based on its fundamental role in induction of cell cycle arrest and apoptosis there is tight regulation of the function of the tumor suppressor p53 in normal cells. In tumor cells, however, the p53 function is frequently inactivated enabling evasion of growth control and outgrowth of malignant cells. In acute myeloid leukemia (AML), the p53 function is rarely disrupted by *TP53* gene mutations, but more often by dysregulation of the nucleolar phosphoprotein nucleophosmin (NPM1) [1], the cellular p53 inhibitor MDM2 [2], the nuclear export protein XPO1/CRM1 [3], or the cytoplasmic retention protein CUL9/PARC [4]. While the *NPM1* gene is commonly mutated in AML, the protein levels of MDM2, XPO1 and CUL9 are frequently elevated in AML cells [5]. In addition to p53 inactivation, there may be activation of oncogenes in the leukemic cell. A key oncogene in AML is the FMS-kinase 3 (*FLT3*) gene. This growth factor receptor is overexpressed in the majority of AML cells [6] or constitutively active in AML cells with FLT3 mutation, observed in up to 30% of AML patients [7]. FLT3 receptor signaling promotes cell survival and prevents apoptosis via activation of the PI3K-PDK1-AKT kinase cascade and via RAS GTPases to the MAP kinase cascade (MEK-ERK-MNK) [8,9]. FLT3-ITD is a constitutively active FLT3 receptor signaling via PI3K-PDK1-AKT, via RAS-RAF-MEK-ERK and via STAT5 [10]. Kinase function can readily be blocked and a number of kinase inhibitors with varying specificity for kinases including the FLT3 and MAP kinases are being tested in preclinical and clinical cancer trials [7,11]. 

Idasanutlin is an MDM2 inhibitor which has been tested in preclinical studies in solid tumors [12,13] and in hematological malignancies including AML [14]. Cobimetinib is a highly selective MEK1 inhibitor studied as a targeted therapy in BRAF and NRAS mutant melanoma [15], and it has been tested in preclinical studies in NRAS mutant AML [16]. In this study, we investigated both the MDM2 antagonist idasanutlin and the MEK1 inhibitor cobimetinib in AML cells in order to identify a potentially effective treatment for subgroups of AML patients unfit for intensive chemotherapy. The study might provide the rationale for initiating a clinical study in TP53wt NK AML evaluating this treatment combination.

## 2. Results

### 2.1. Synergistic Effects on Cell Viability in AML Cell Lines Treated with the MDM2 Inhibitor Idasanutlin and the MEK Inhibitor Cobimetinib

To determine the sensitivity of AML cells to idasanutlin and cobimetinib we tested the cell viability of a variety of AML cell lines in in vitro cytotoxicity assays. The cell lines covered the major molecular AML subtypes characterized by the *FLT3*-ITD and *FLT3* wild type, *NPM1* mutant and wild type, *TP53* mutant and wild type as well as *RAS* mutant and wild type genes (Table 1). 

**Figure 1:**
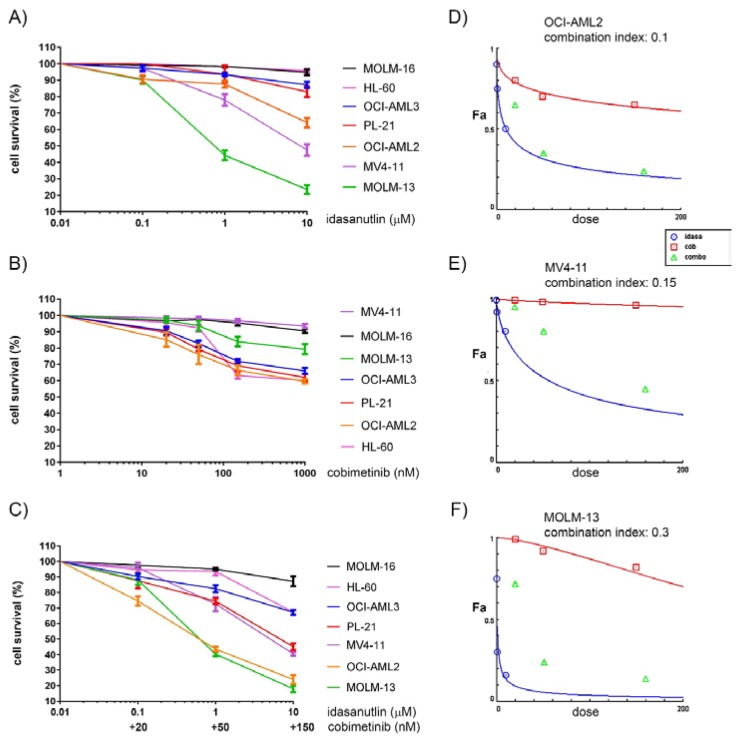
Sensitivity of AML cells to idasanutlin and cobimetinib. Cell viability in AML cell lines treated for 24 h with increasing dosages of idasanutlin (**A**) or cobimetinib (**B**) or in direct combination (**C**). Dose-effect curves in OCI-AML2 (**D**), MV4-11 (**E**) and MOLM-13 cells (**F**) treated with idasanutlin and cobimetinib alone or in combination. Fa indicates fraction of affected cells. Combination indexes were calculated on compusyn software (version 1.0; ComboSyn, Inc. Paramus, NJ, USA).

We observed considerable differences in the AML cells lines with respect to idasanutlin and cobimetinib sensitivity (Figure 1A,B). MOLM-13 *(FLT3-*ITD) cells were most sensitive to idasanutlin with 50% loss of cell viability to 0.5 µM idasanutlin in 24 h. At 10 µM idasanutlin, MV4-11 (*FLT3*-ITD) and OCI-AML2 (*FLT3*wt) had 50% loss of cell viability whereas OCI-AML3 (*FLT3*wt), PL-21 (*FLT3*-ITD) and MOLM-16 (*TP53*mut) cells only lost 10% of cell viability. With respect to cobimetinib OCI-AML2 (*RAS*wt), PL-21 (*KRAS*mut), HL-60 (*NRAS*mut) and OCI-AML3 (*NRAS*mut) cell lines were susceptible to a 30% loss of cell viability to 150 nM cobimetinib in 24 h. At 1 µM cobimetinib, MOLM-13 (*RAS*wt) cells had 20% loss of cell viability while MV4-11 (*RAS*wt) and MOLM-16 (*RAS*wt) cells only lost 10% of cell viability. To determine the optimal regime for combination treatments we tested the effects of sequential versus direct combination treatment with idasanutlin and cobimetinib. Sequence of application was not important as idasanutlin pretreatment followed by cobimetinib treatment had the same effect on cell viability as cobimetinib pretreatment followed by idasanutlin treatment. Moreover, both sequential treatments had nearly the same effect on cell viability as direct combination treatment (Figure S1). In the direct combination treatment with idasanutlin and cobimetinib, we again observed considerable differences in the responses of the tested AML cell lines (Figure 1C). OCI-AML2, MV4-11, MOLM-13, PL-21 and OCI-AML3 cells showed synergistic responses with combination indexes of 0.1, 0.15, 0.3, 0.55 and 0.8, respectively (Figure 1D–F). The addition of idasanutlin did not lead to an enhanced cytotoxic response of TP53 deficient HL-60 cells to cobimetinib and TP53 mutant MOLM-16 cells were resistant to both compounds in the tested concentrations (Figure 1C). In summary, OCI-AML2 cells were susceptible to idasanutlin and cobimetinib as single compounds with strong synergistic effects in the combined treatment. Susceptibility of MOLM-13 and MV4-11 cells was substantial towards idasanutlin, but marginal to cobimetinib, with synergistic effects in the combination treatment. Susceptibility of RAS mutated cells PL-21 and OCI-AML3 was substantial towards cobimetinib, but marginal to idasanutlin, with moderate synergistic effects in the combination treatment. With substantial reduction of cell viability in sensitive AML cell lines at 0.5 to 1 µM idasanutlin and 50 to 100 nM cobimetinib, we chose to test molecular effects in AML cell lines treated at these concentrations of inhibitors. 

### 2.2. Inhibition of Cell Cycling and Induction of Apoptosis by Idasanutlin and Cobimetinib 

**Figure 2:**
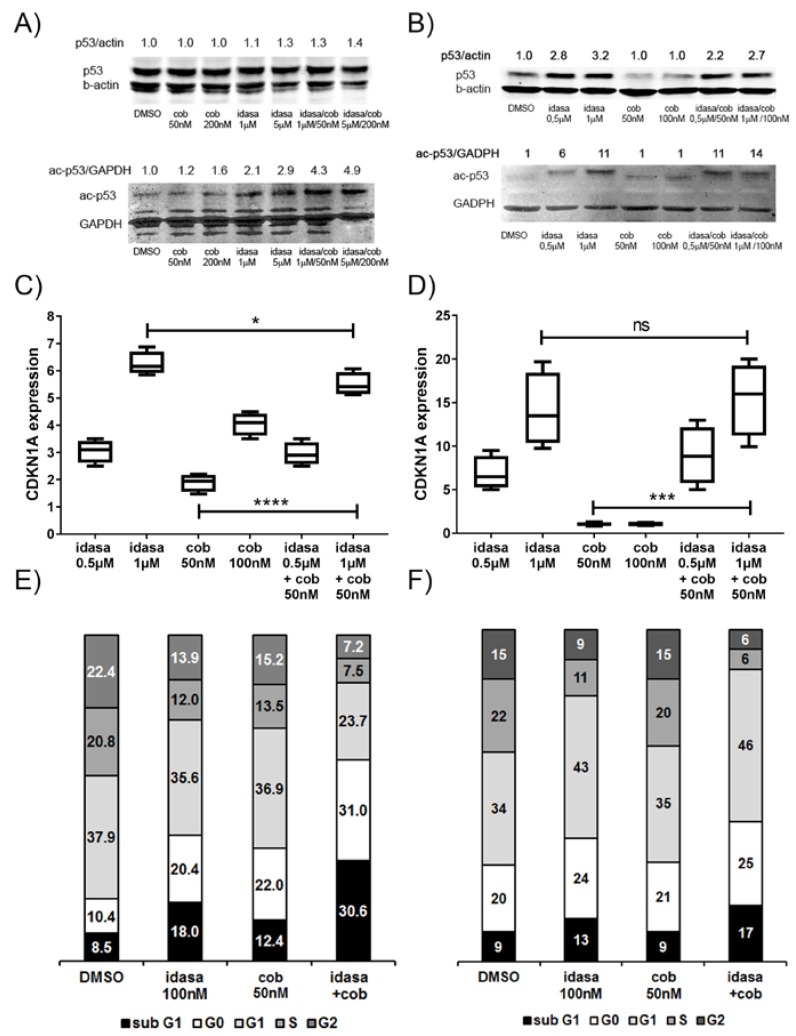
Induction of cell cycle arrest, cell cycle exit and cell death in AML cells treated with idasanutlin and cobimetinib. Induction of tumor suppressor protein p53 in OCI-AML2 (**A**) and MOLM-13 cells (**B**) treated for 24 h with the indicated amounts of idasanutlin and cobimetinib. Induction of the cell cycle inhibitor *CDKN1A* gene in OCI-AML2 (**C**) and MOLM-13 cells (**D**). Relative quantitation of *CDKN1A* mRNA normalized to *GAPDH*. Data are presented as fold induction of treated versus untreated, with CDKN1A expression in DMSO treated cells = 1. Induction of cell cycle arrest, cell cycle exit and cell death in the AML cell lines OCI-AML2 (**E**) and MOLM-13 (**F**) treated for 24 h with single compounds or with combination treatment. Cells were grouped into defined cell cycle phases by DNA content and Ki-67 expression levels with subG1 (<2n), G0 (2n, Ki67 low), G1 (2n, Ki67 high), S (2n–4n), and G2 (4n). Significance is denoted for *p* < 0.05 (*); *p* < 0.0005 (***); *p* < 0.0001 (****); *p* > 0.05 (ns).

To confirm p53 activation by MDM2 inhibition we determined the expression levels of the tumor suppressor protein p53, of the activated acetylated p53 protein and of the p53 target gene *CDKN1A* in AML cells treated for 24 h with single compounds and with combined treatment. Protein p53 levels were rather high in untreated OCI-AML2 cells with a 50% increase of protein level and a three-fold induction of acetylated p53 in idasanutlin treated cells (Figure 2A). MOLM-13 protein p53 levels were induced three-fold and acetylated p53 levels over ten-fold in idasanutlin treated cells (Figure 2B). Moreover, there was a four-fold p53 protein induction in idasanutlin treated MV4-11 cells (Figure S2) confirming that p53 activation is a general consequence of treatment with the MDM2 inhibitor idasanutlin. *CDKN1A* gene expression was induced six-fold in OCI-AML2 (Figure 2C), twelve-fold in MOLM-13 cells treated with 1 µM idasanutlin (Figure 2D) and ten-fold in MV4-11 (Figure S2). Interestingly, there was also an induction of *CDKN1A* expression in OCI-AML2 and MV4-11 cells treated with 50 nM cobimetinib, but not in MOLM13 cells, indicating that MEK inhibition can lead to p53 activation, at least in some AML cells. Acetylated p53 levels were maximal in AML cells treated with combinations of idasanutlin and cobimetinib. *CDKN1A* expression was induced five-fold in OCI-AML2, sixteen-fold in MOLM-13 and MV4-11 cells, indicating a negative effect of cobimetinib on the idasanutlin induced *CDKN1A* gene expression in OCI-AML2 cells and a positive effect in MOLM-13 and MV4-11 cells. Indeed the combination index for the effect of the combined treatment on CDKN1A expression was calculated at 1.6 in OCI-AML2 cells confirming an antagonistic effect of cobimetinib on idasanutlin induced CDKN1A expression, and 0.8 in MV4-11 cells indicating a moderate synergistic effect in this context. 

To demonstrate the induction of cell cycle arrest in AML cells with elevated levels of the cell cycle inhibitor CDKN1A, the cell cycle was analyzed by DNA content measurement employing flow cytometry in OCI-AML2 (Figure 2E and Figure S4A) and MOLM-13 cells (Figure 2F and Figure S4B) treated with single compounds or combined treatment. As treatments with 1 µM idasanutlin and 50 nM cobimetinib induced substantial cell death in the in vitro cytotoxicity assays, we applied the compounds at lower concentrations in the flow cytometry assays. This entailed treating OCI-AML2 cells with 100 nM idasanutlin and induced cell cycle exit and cell death illustrated by an increase of resting cells (G0) from 10% to 20% and an increase of dead cells (sub-G1) from 8% to 18% with a concomitant reduction in the proliferating cells (S and G2) (Figure 2C). Treating OCI-AML2 cells with 50 nM cobimetinib had similar effects with a greater emphasis on cell cycle exit and minor induction of cell death. Compared to single compounds, idasanutlin and cobimetinib together led to a further increase in cell cycle exit and cell death, with over 30% resting and over 30% dead cells with concomitant reduction in proliferating cells (G1, S and G2). In contrast to OCI-AML2 cells, 50 nM cobimetinib had no effect on MOLM-13 cells, whereas idasanutlin treatment led to an 11% increase in G1 and a 4% increase in G0 phase cells with a concomitant reduction of S- and G2-phase cells indicating G1cell cycle arrest (Figure 2D). The same 24 h treatment with idasanutlin led to a G1 arrest in MOLM-13 cells, and an increase of G0 and subG1 fractions in OCI-AML2 cells. As cells exit the cycle at G1 to enter G0, the increase in G0 and subG1fractions in OCI-AML2 was necessarily preceded by a G1 arrest. Compared to MOLM-13 OCI-AML2 cells showed a faster response in the induction of G1 arrest and cell cycle exit.

**Figure 3:**
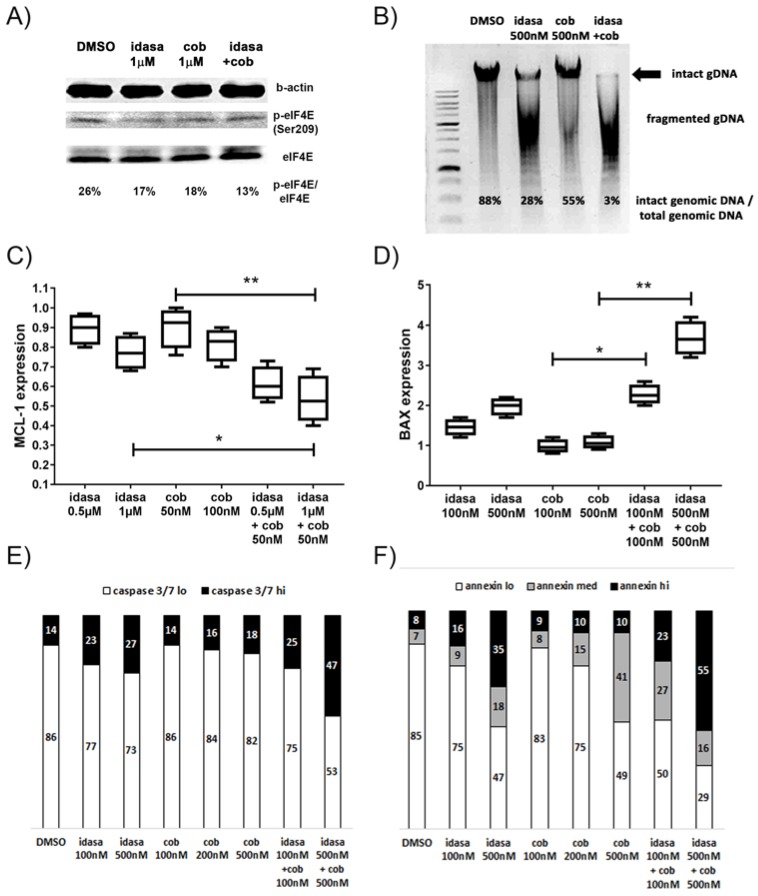
Induction of apoptosis in AML cells treated with idasanutlin and cobimetinib. Reduced levels of activated translation initiation factor phospho-eIF4E (**A**), induction of apoptotic DNA fragmentation (**B**), reduced mRNA levels of the apoptosis inhibitor *MCL1* gene (**C**), induced mRNA levels of the pro-apoptotic BAX gene (**D**), induced caspase 3/7 activity (**E**), increase in annexin positive apoptotic MOLM-13 cells (**F**) treated with the indicated amounts of idasanutlin (idasa) and cobimetinib (cob). Cells were grouped into viable (annexin lo), early apoptotic (annexin med), and late apoptotic (annexin hi) cells. Significance is denoted for *p* < 0.05 (*); *p* < 0.005 (**).

In addition to *CDKN1A* induction, p53 activation due to MDM2 or MEK inhibition also led to reduced phosphorylation of eIF4E, *BAX* gene induction, *MCL1* gene repression and eventually to cell death by apoptosis. Differences in eIF4E phosphorylation and in apoptotic DNA fragmentation were detectable only at higher concentrations of cobimetinib. The levels of phosphorylated eIF4E fell from 26% to 17% in MOLM-13 cells treated with 1 µM idasanutlin or 1 µM cobimetinib with the lowest levels of 13% in the combined treatment (Figure 3A). The pattern of fragmented DNA typical for apoptosis and a concomitant reduction of the amount of intact genomic DNA was present in MOLM-13 cells treated with 0.5 µM idasanutlin or 0.5 µM cobimetinib, with greatest reduction in the combined treatment, again indicating a synergistic effect of idasanutlin and cobimetinib on apoptosis induction (Figure 3B). *MCL1* gene expression in the 1 µM idasanutlin treated cells was at 75% compared to control DMSO treated cells, at 90% in the 50 nM cobimetinib treated cells and at 55% in the combination treatment (Figure 3C). With a combination index of 0.45 the inhibitory effect of cobimetinib and idasanutlin on *MCL1* gene expression in MOLM-13 cells appeared to be synergistic. There was induction of *BAX* gene expression and caspase 3/7 activities in MOLM-13 and OCI-AML2 cells treated with idasanutlin, with further induction in combination treated cells (Figure 3D,E, Figures S3A and S5A). There was a dose dependent increase in the number of annexin positive cells in MOLM-13 and OCI-AML2 cells treated with idasanutlin and cobimetinib (Figure 3F, Figures S3B and S5B). The maximum number of annexin positive cells was present after combined treatment indicating a synergistic effect of idasanutlin and cobimetinib on apoptosis induction. 

**Figure 4:**
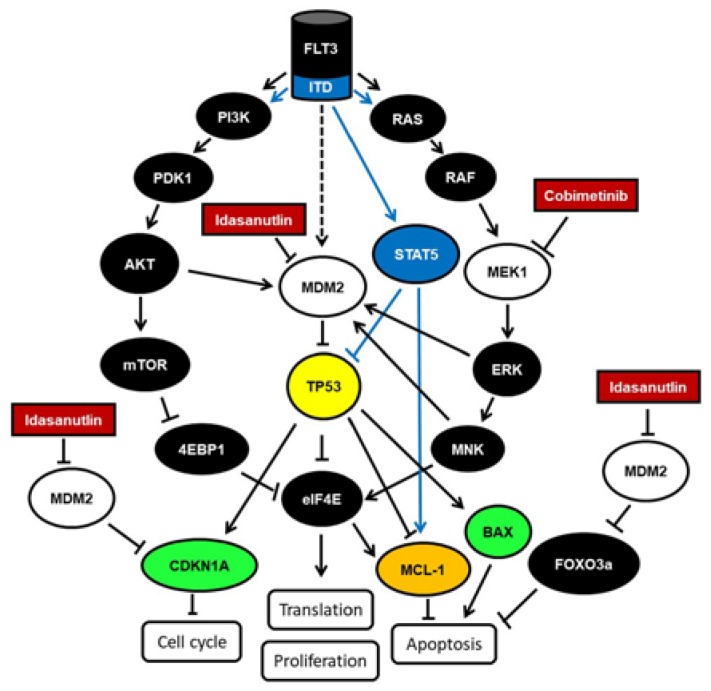
Schematic representation of the FLT3 signaling pathways and downstream effects. FLT3 is a growth factor receptor signaling via PI3K-PDK1-AKT and via RAS-RAF-MEK-ERK leading to cell growth and proliferation involving p53 inhibition. Idasanutlin and cobimetinib inhibit MDM2 and MEK1 leading to reactivation of the tumor suppressor p53 and to induction of *CDKN1A* and BAX gene expression as well as inhibition of *MCL1* gene expression and in consequence to induction of cell cycle arrest and apoptosis. FLT3-ITD is a constitutively active FLT3 receptor signaling via PI3K-PDK1-AKT, via RAS-RAF-MEK-ERK and via STAT5.

In summary, these data suggest that AML cells treated with the MDM2 inhibitor idasanutlin and the MEK1 inhibitor cobimetinib induced *CDKN1A* gene expression and cell cycle arrest, cell cycle exit and cell death, reduced levels of activated eIF4E protein, induced *BAX* gene expression, suppressed *MCL1* gene expression, induced caspase3/7 activity and triggered apoptosis as summarized in Figure 4. One or the other downstream effects might be preferentially induced in different AML cells, depending on the differential activation of p53 by acetylation and methylation. Acetylated p53 may have an increased ability to transcribe BAX, but not CDKN1A, resulting in a preferential activation of the apoptotic pathway over cell cycle arrest, while methylated p53 may have an increased ability to transcribe CDKN1A, resulting in a preferential induction of cell cycle arrest [17,18].

### 2.3. Varying Sensitivity of AML Patient Cells to Treatment with the MDM2 Inhibitor Idasanutlin and the MEK Inhibitor Cobimetinib

Considering the wide range of responses observed in AML cell lines to the combination treatment with idasanutlin and cobimetinib, we hypothesized that susceptibility to this treatment would be different among genetically defined AML subgroups. We performed in vitro cytotoxicity assays using mononuclear cells isolated from peripheral blood or bone marrow of a small panel of AML patients (Table 2). The blast cells covered the major molecular subtypes including *FLT3*-ITD and *FLT3* wild type, *NPM1* mutant and *NPM1* wild type, as well as *TP53* mutant and *TP53* wild type cells. The majority of the blast cells carried wild type RAS genes with only two NRAS mutations present in the twenty-seven samples.

**Figure 5:**
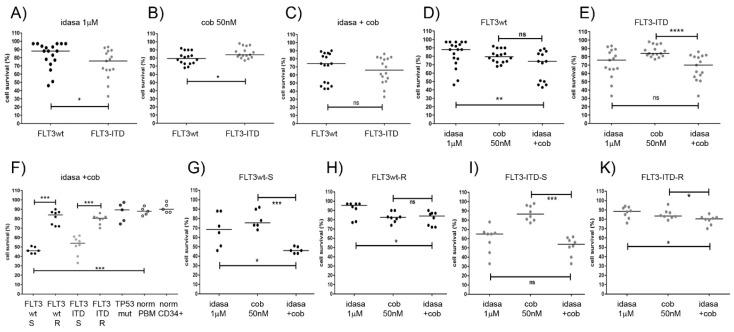
Effects of idasanutlin and cobimetinib treatment on AML patient and normal blood samples. Cell viability assessments in AML patient cells treated for 24 h with idasanutlin and cobimetinib alone or in combination are depicted for the genetic subgroups according to the *FLT3* status (**A**–**E**), for the response subgroups according to *FLT3* status and sensitivity to the combination treatment (**F**), for the response subgroups FLT3wt-S (**G**), FLT3wt-R (**H**), FLT3ITD-S (**I**) and FLT3ITD-R (**K**). In our definition, sensitive AML cells had less than 70% cell viability after 24 h treatment with 1 µM idasanutlin and 50 nM cobimetinib. * indicates significant *p*-values in Mann-Whitney test. AML blast cells were grouped according to molecular subtypes (*FLT3/TP53*) and according to response rates. Normal samples were grouped into peripheral blood mononuclear cells (PBM) and normal HSCs (CD34+). Significance is denoted for *p* < 0.05 (*); *p* < 0.005 (**); *p* < 0.0005 (***); *p* < 0.0001 (****); *p >* 0.05 (ns).

The responses to idasanutlin and cobimetinib varied among the tested AML samples (Table S1). In order to find correlations of response to genotype we initially grouped the NK-AML samples according to FLT3 status. In grouped analysis, we observed that *FLT3*-ITD cells were more susceptible to idasanutlin (Figure 5A) and less susceptible to cobimetinib (Figure 5B) compared to *FLT3*wt cells, whereas there was no significant difference in the sensitivity towards the combination treatment in *FLT3*wt and *FLT3*-ITD cells (Figure 5C). However, adding cobimetinib enhanced effects of idasanutlin on *FLT3*wt cells (Figure 5D), but not on *FLT3*-ITD cells (Figure 5E). 

Half of the AML samples were sensitive to idasanutlin, with less than 80% cell viability at 24 h treatment with 1 μM compound, comprising six *FLT3*wt and eight *FLT3*-ITD AML samples. *NPM1* status did not correlate to idasanutlin sensitivity as half of the *FLT3*wt and half of the *FLT3*-ITD samples were the *NPM1* wild type. In contrast to idasanutlin all AML cells including the *TP53* mutated samples showed some response to cobimetinib, Nine AML samples were sensitive to cobimetinib with less than 80% viability after 24 h treatment with 50 nM of the compound, of which six were *FLT3*wt, one *FLT3-*ITD and two *TP53* mutated AML. 

Half of the AML samples were susceptible to the combination treatment with 1 μM idasanutlin and 50 nM cobimetinib including five *FLT3*wt and seven *FLT3-*ITD NK-AML samples with 30% to 60% cell viability after 24 h treatment (Table S1). The other half of the AML samples were much less affected by the combination treatment and included six *FLT3*-ITD and six *FLT3*wt NK-AML as well as three *TP53*mut samples with complex karyotypes (Table S1). In order to define response subgroups, the AML samples were separated into NK-AML and CK-AML and the NK-AML were grouped according to *FLT3* status as well as sensitivity to combination treatment (FLT3wt-S, FLT3wt-R, FLT3ITD-S, FLT3ITD-R) (Figure 5F). Cells least affected by single agent and combination treatment were normal peripheral blood monocytes and CD34 positive selected bone marrow cells. As in OCI-AML2 cells, the response of *FLT3*wt-S AML patient cells to 1 µM idasanutlin was significantly enhanced by the addition of 50 nM cobimetinib (Figure 5G). Similar to OCI-AML3 cells, *FLT3*wt-R AML cells responded to cobimetinib (Figure 5H). Moreover, as in MOLM-13 cells, the addition of 50 nM cobimetinib did not significantly enhance the response of *FLT3*-ITD-S patient cells to 1 μM idasanutlin (Figure 5I) whereas *FLT3*-ITD-R patient cells showed minor response to both compounds (Figure 5K). These data suggest that about half of the TP53wt NK-AML were susceptible to a combination treatment with idasanutlin and cobimetinib whereas *TP53*mut AML cells as well as normal peripheral blood monocytes and normal CD34 positive selected bone marrow cells were minimally affected. 

### 2.4. Correlation of Cellular FLT3 and MDM2 Protein Levels with Susceptibility to Idasanutlin and Cobimetinib

**Figure 6:**
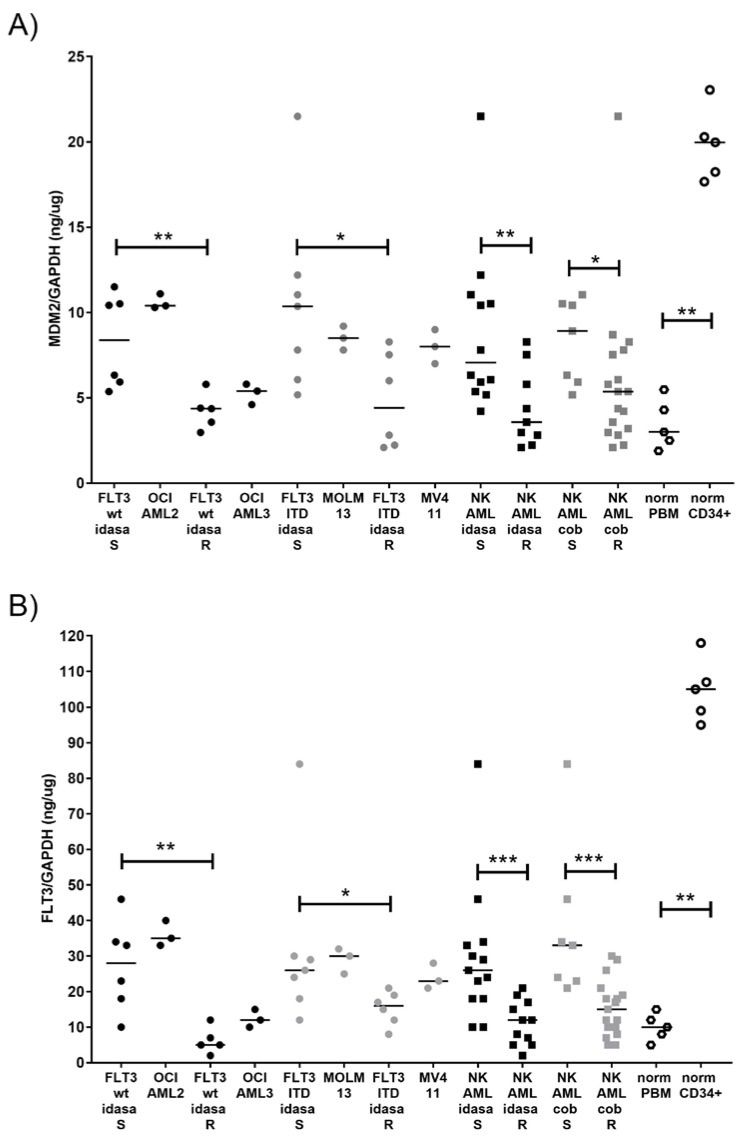
MDM2 and FLT3 protein levels correlate with treatment response in AML cell lines and patient samples. MDM2 (**A**) and FLT3 (**B**) protein levels as determined by ELISA and normalized to GAPDH. AML blast cells were grouped according to molecular subtypes (FLT3/TP53) and according to response rates. Sensitive AML cells were defined as having less than 80% cell viability after 24 h treatment with 1 μM idasanutlin (idasa-S) or 50 nM cobimetinib (cob-S). * indicates significant *p*-values in Mann-Whitney test. FLT3wt (black circles), FLT3-ITD (grey circles), NK-AML idasanutlin response (black squares), NK-AML cobimetinib response (grey squares), normal PBM (hexagons) and normal CD34+ cells (open circles). Significance is denoted for *p* < 0.05 (*); *p* < 0.005 (**); *p* < 0.0005 (***).

As there was no apparent correlation of *NPM1* gene status with idasanutlin sensitivity in *FLT3*wt AML cells, we hypothesized that susceptibility to the MDM2 inhibitor may depend on the abundance of the target protein in the AML cells. We therefore determined the MDM2 protein levels in AML blast cells as well as in normal peripheral blood mononuclear cells (PBMC) and in normal hematopoietic stem cells (HSC). MDM2 protein levels ranged from 2 ng/µg to 25 ng/µg (MDM2/GAPDH), with highest levels observed in the stem cell samples. Low MDM2 levels were present in normal PBMC as well as in idasanutlin-resistant AML cells. Compared to PBMC and idasanutlin-resistant cells, idasanutlin-sensitive NK-AML cells had elevated MDM2 protein levels with median levels of 7 versus 3.5 ng/µg (Figure 6A). The median MDM2 levels in sensitive and resistant subgroups were 8 versus 4 ng/µg, and 10 versus 3 ng/µg in FLT3wt and FLT3-ITD NK-AML respectively. Surprisingly, cobimetinib-sensitive AML cells also had elevated MDM2 levels with 8 versus 5 ng/µg, indicating that NK AML cells with elevated MDM2 levels may be particularly susceptible to the combined treatment with idasanutlin and cobimetinib. 

As FLT3 signaling activity may correlate to MDM2 protein levels and MEK activity, we also determined the FLT3 protein levels in all samples. Indeed, there was a strong correlation of FLT3 and MDM2 levels (Figure S6). FLT3 levels ranged from 1 to 120 ng/µg (FLT3/GAPDH) with highest levels in the normal CD34 positive stem cells (Figure 6B). Low FLT3 levels were present in normal PBMC as well as in idasanutlin-resistant AML cells. Compared to PBMC and idasanutlin-resistant cells, idasanutlin-sensitive NK-AML cells had elevated FLT3 protein levels with median levels of 25 versus 12 ng/µg. The median FLT3 levels in sensitive and resistant subgroups were 28 versus 4 ng/µg, and 26 versus 13 ng/µg in FLT3wt and FLT3-ITD NK-AML, respectively. Cobimetinib sensitive AML cells also had elevated FLT3 levels (32 versus 16 ng/µg) indicating that NK AML cells with elevated FLT3 levels may be particularly susceptible to the combined treatment with idasanutlin and cobimetinib.

## 3. Discussion

The aim of this study was to identify AML subgroups with particular sensitivity to the combined treatment with the MDM2 antagonist idasanutlin and the MEK1 inhibitor cobimetinib. We assessed the two inhibitors as single agents and in combination treatments in a variety of AML cell lines and in a variety of AML patient leukemic cells for their ability to induce apoptosis and cell death. As MDM2 inhibition can only be effective in a *TP53* wild type setting *TP53* mutated AML cells were, in fact, not susceptible to idasanutlin treatment. Interestingly, however, only half of the *TP53*wt AML cells were susceptible to idasanutlin. Idasanutlin had a greater effect on *FLT3*-ITD than on *FLT3*wt cells, while the presence of a *NPM1* mutation had no effect on MDM2 inhibitor sensitivity. Similar correlations have previously been described for other MDM2 inhibitors [19]. Sensitivity to idasanutlin treatment appeared to be limited to AML subgroups characterized by MDM2 overexpression, whereas idasanutlin resistance was observed in AML subgroups characterized by MDMX overexpression [20].

It was to some extent unexpected that sensitivity to the MDM2 inhibitor was not affected in the presence of mutated *NPM1* (NPMc) which has lost the ability to stabilize the tumor suppressor p53 in the nucleus and may inhibit p53 by cytoplasmic retention. Apparently, the presence of reduced levels of normal NPM1 protein can provide adequate stabilization of p53 to induce cell cycle arrest and apoptosis in idasanutlin treated *NPM1* mutated AML cells. However, we found that both FLT3 and MDM2 expression levels appear to be adequate biomarkers to predict response of AML cells to idasanutlin. Supporting evidence for MDM2 expression as a predictive marker came from a phase I study with idasanutlin where AML patients with elevated MDM2 protein expression had improved outcomes [21]. The molecular mechanisms underlying the correlation of MDM2 protein levels and response to MDM2 inhibitors may be related to the phenomenon of oncogene addiction where the tumor relies on a dominant oncogene or mechanism for growth and survival, so that inhibition of this specific oncogene/pathway is sufficient to halt the neoplastic phenotype [22,23].

Similar to idasanutlin, not all AML cells were sensitive to the MEK1 inhibitor cobimetinib. Notably, RAS mutated cells were more susceptible to cobimetinib than RAS wild type cells. Here the molecular mechanism is, in all likelihood, an addiction to the RAS oncogene. In addition to RAS- mutated cells, *FLT3*wt cells were generally more susceptible to cobimetinib than *FLT3*-ITD cells. Most likely, wild type FLT3 receptor signaling is more dependent on MEK activity than FLT3-ITD receptor signaling. *FLT3*-ITD mutants induce auto-phosphorylation of the receptor, interleukin 3-independent growth and a strong STAT5 and mitogen-activated protein kinase (MAPK) activation. In contrast to the *FLT3*-ITD mutants, ligand stimulated FLT3 wild-type receptors activate AKT and MAPK, but not STAT5 [10]. Thus, the acquisition of *FLT3*-ITD ensures leukemic stem cell survival by up-regulating MCL-1 gene expression via constitutive STAT5 activation that is independent of wild-type FLT3 signaling [24]. 

Surprisingly, susceptibility to cobimetinib did not strictly correlate to the mutational status of *RAS* genes, but, unexpectedly, to FLT3 and MDM2 protein levels. *RAS* mutant cell lines OCI-AML3, HL-60 and PL-21 as well as RAS mutated AML patient samples showed sensitivity to cobimetinib, as well as AML cells with normal *RAS* genes like OCI-AML2 and a subset of the AML patient samples with normal RAS status. In studies with other MEK inhibitors there was also no strict correlation of the responses with the mutational or phosphoprotein status of RAS or RAF [25]. However, it was described that different compensatory RAS effectors including PI3K can mediate resistance to MEK inhibitors. In any case, our data indicate that FLT3 and MDM2 expression may be adequate biomarkers to predict response of AML cells not only to idasanutlin, but also to cobimetinib. 

How can activated RAS lead to p53 inhibition in AML cells? RAS can have opposing effects on p53 [20]. Levels of p53 are determined by opposing effects of ARF and MDM2. In a variety of primary cells activated oncogenic RAS leads to p53 induction via induction of the MDM2 inhibitor ARF [20]. In AML cells, activated (normal) RAS can induce MDM2 via MEK and ERK and thereby inhibit p53. Perhaps activated RAS is unable to induce ARF in AML cells? Indeed NPM1 overexpression can antagonize ARF function while increasing its nucleolar localization [26] and ARF appears to be inactivated in NPM1 mutated AML cells [27]. 

Susceptibility to the combination treatment with cobimetinib and idasanutlin was present in AML cells defined by normal karyotype (NK) and wild type status of the *TP53* gene with elevated FLT3 and MDM2 protein levels. The correlation of FLT3 and MDM2 levels and sensitivity to both idasanutlin and cobimetinib in AML cells may be based on these molecular mechanisms: MDM2 levels can be induced by FLT3 and FLT3-ITD via AKT, but also by activated RAS via MEK and ERK [28], while MDM2 mRNA export from the nucleus is dependent on MEK and MNK1 activity [29]. Thus, elevated MEK, ERK and MNK1 activities induce high levels of MDM2 protein leading to p53 inhibition and cell proliferation in AML cells. Treatment with cobimetinib and idasanutlin may have synergistic effects on viability of AML cells with elevated FLT3 and MDM2 levels by concomitant inhibition of MEK and MDM2. 

Synergistic effects on cell viability with idasanutlin and cobimetinib were present particularly in OCI-AML2, MV4-11 and MOLM-13 cells and in several *FLT3*wt and *FLT3-*ITD patient samples with elevated FLT3 and MDM2 protein levels. Synergistic effects were present in MV4-11 cells, particularly regarding *CDKN1A* gene induction, and in MOLM-13 cells, particularly regarding *MCL1* gene inhibition and apoptosis induction. As there is independent regulation of *MCL-1* gene expression by p53 and by MEK, inhibiting both MDM2 and MEK can have synergistic effects on *MCL1* gene expression. In contrast, cobimetinib treatment had negative effects on the idasanutlin induced *CDKN1A* gene expression in OCI-AML2 cells. Single compound treatment with idasanutlin or with cobimetinib can induce *CDKN1A* gene expression in OCI-AML2 cells indicating that regulation of *CDKN1A* gene expression by p53 and MEK is dependent in this cell line. Consequently, idasanutlin and cobimetinib can separately induce the downstream effect of *CDKN1A* induction such as G1 cell cycle arrest followed by cell cycle exit in OCI-AML2 cells, with an enhanced effect in the combination treatment. 

Synergistic effects on cell viability with idasanutlin and cobimetinib were present independent of the sequence of application, indicating that the order of target inhibition for MEK1 and MDM2 was not important. Sequential application of idasanutlin and cobimetinib had the same effects on cell viability as direct combination treatment. Pretreatment with one inhibitor did not enhance the susceptibility of AML cells to the second inhibitor. This allows several treatment regimens available for testing in a possible future clinical trial. 

Our data indicate that the combination of idasanutlin and cobimetinib may be an effective and specific treatment to target NK-AML with wild type *TP53* and elevated FLT3 and MDM2 levels. Within a clinical trial, FLT3 and/or MDM2 protein levels in the blast cells may allow patient stratification; therefore, FLT3 and MDM2 cytometry with peripheral blood leukocytes may be required to support treatment decisions. In the future, it may be feasible to do a full array of treatment response assays including p53 target gene induction in a high-speed setup before starting therapy. NK-AML with low FLT3 and MDM2 levels may be treated by targeting other regulators of the p53 function frequently overexpressed in AML like MDMX, XPO1 and CUL9/PARC [5]. Combination treatments with more than two targeted compounds may increase therapeutic specificity and efficacy and become prevalent in the near future. Moreover, AML patients fit for intensive therapy may profit from combination treatments with targeted compounds and conventional chemotherapy.

## 4. Materials and Methods

### 4.1. Cell Lines

OCI-AML2 (*FLT3*wt, *TP53*wt), OCI-AML3 (*FLT3*wt, *NPM1*L287fs, *NRAS*Q61L, *TP53*wt), MOLM-13 (*FLT3*ITD, t(9;11), *TP53*wt), MV4-11 (*FLT3*ITD, t(4;11), *TP53*wt), PL-21 (*FLT3P336L*, *KRAS*A146V, *TP53*P36fs), HL60 (*FLT3*wt, *TP53*del) and MOLM-16 (*FLT3*wt, *TP53*V173M, *TP53*C238S) cell lines were supplied by the Leibniz Institute DSMZ, German Collection of Microorganisms and Cell Cultures (Braunschweig, Germany). AML cells were grown in RPMI 1640 (SIGMA-ALDRICH, St. Louis, USA) supplemented with 20% fetal bovine serum (FBS, Biochrom GmbH, Germany). Cells were passaged for a minimal number of times between thawing and use in the described experiments. 

### 4.2. Patient Samples

Mononuclear cells of AML patients diagnosed and treated at the University Hospital, Bern, Switzerland between 2015 and 2016 were included in this study. Informed consent of all patients in the study was obtained according to the Declaration of Helsinki, and the studies were approved by decisions of the local ethics committee of Bern, Switzerland. Mutational screenings for *FLT3, NPM1, TP53* genes and conventional karyotype analysis of at least 20 metaphases were performed for each patient. Peripheral blood mononuclear cells (PBMCs) and bone marrow mononuclear cells (BMMCs) were collected at the time of diagnosis before treatment start. 

### 4.3. Methods

Description of the methods in the supplements: Measurement of mRNA expression by qPCR, DNA fragmentation assay, Flow cytometry, Imaging cytometry, Measurement of protein levels by Western Blot and Enzyme-linked immunosorbent Assay (ELISA).

## 5. Conclusions

AML cells with normal karyotype (NK) and wild type status of TP53 with elevated FLT3 and MDM2 protein levels emerge to be susceptible to the combined treatment with the MEK1 inhibitor cobimetinib and the MDM2 inhibitor idasanutlin. FLT3 and MDM2 are biomarkers for treatment response to idasanutlin and cobimetinib in AML. This preclinical study might provide the rationale for initiating a clinical study in TP53wt NK-AML patients unfit for intensive chemotherapy evaluating this treatment combination.

## Figures and Tables

**Table 1:** Acute myeloid leukemia (AML) cell lines and patient samples

ID	FLT3	TP53	Mutated Genes
OCI-AML2	wt/A680V	wt	*DNMT3A*
FLT3wt-S (5)	wt	wt	*NPM1 (3) NRAS (1)*
MOLM-13	ITD	wt	*MLL-AF9 mTOR*
FLT3-ITD-S (7)	ITD	wt	*NPM1 (4)*
MV4-11	ITD	wt	*MLL-AF4*
FLT3-ITD-R (6)	ITD	wt	*NPM1 (2) RUNX1 (2)*
PL-21	wt/P336L	wt/P36fs	*KRAS*
HL-60	wt	del	*NRAS*
FLT3wt-R (5)	wt	wt	*NPM1 (2) DNMT3A (1)*
MOLM-16	wt	mut	*MLL V1368L*
TP53mut (3)	wt	mut	*PTPN11 (1) NRAS (1)*

AML cell lines (grey) and AML patient samples (white); grouped according to FLT3 and TP53 status and response to combination treatment (number of samples).

**Table 2:** AML cohort study samples.

Clinical Characteristic		Absolute Number	Relative Number (%)
AML		27	100
FAB classification	M0	0	0
	M1	6	22
	M2	7	26
	M3	0	0
	M4	6	22
	M5	8	30
	M6	0	0
	M7	0	0
Pathogenesis	de novo	26	96
	relapse	1	4
Molecular Analysis	FLT3wt/NPM1wt	15	56
	FLT3-ITD	12	44
	NPM1mut	12	44
	FLT3-ITD and NPM1mut	6	22
Cytogenetic Analysis	normal karyotype (NK)	20	74
	−5, −5q	0	0
	−7, −7q	3	11
	inv(16)	1	4
	t(8;21)	0	0
	t(15;17)	0	0
	complex karyotype (CK)	3	11
Risk Category *	favorable	6	22
	intermediate	6	22
	adverse	15	56

* Favorable risk comprised t(8;21), t(15;17), inv(16) and NK with NPM1mut, FLT3-ITD negative; intermediate risk comprised NK without mutations or with FLT3-ITD and NPM1mut; adverse risk comprised −5, −5q, −7, −7q, NK with FLT3-ITD, complex karyotype.

## Supplementary Materials

The following are available online at http://www.mdpi.com/2072-6694/10/6/170/s1, Supplemental Methods: Cytotoxicity assays, Measurement of mRNA expression by qPCR, DNA fragmentation assay, Flow cytometry, Measurement of protein levels by Western Blot, Enzyme-linked immunosorbent Assay (ELISA), Figure S1: Synergistic effect on cell viability in OCI-AML2 cells treated with idasanutlin and cobimetinib independent of sequence of drug application. Cell viability measurements in cells pretretead for 6 h and treated for 24 h with idasanutlin and cobimetinib in all combinations. Idasanutlin pretreatment followed by cobimetinib treatment had the same effect on cell viability as cobimetinib pretreatment followed by idasanutlin treatment. Moreover, both sequential treatments had nearly the same effect on cell viability as 30 h of direct combination treatment, Figure S2: Induction of the tumor suppressor protein p53 and the cell cycle inhibitor *CDKN1A* gene in MV4-11 cells treated with idasanutlin and cobimetinib. Western blot for p53 and b-actin (**A**) and quantitative PCR for *CDKN1A* mRNA normalized to *GAPDH* mRNA (**B**) in MV4-11 cells treated with the indicated amounts of idasanutlin and cobimetinib, Figure S3: Induction of *BAX* gene expression and apoptosis in OCI-AML2 cells treated with idasanutlin and cobimetinib. Quantitative PCR for *BAX* mRNA normalized to *GAPDH* mRNA (**A**) and imaging cytometry for annexin positive cells (**B**) in OCI.AML2 treated with the indicated amounts of idasanutlin and cobimetinib, Figure S4: Induction of cell cycle exit and cell death in OCI-AML2 and MOLM-13 cells treated with idasanutlin and cobimetinib. Cell cycle analysis by flow cytometry measurements of DNA content (DAPI) and proliferation marker Ki-67 in OCI-AML2 (**A**) and MOLM-13 (**B**) treated with the indicated amounts of idasanutlin and cobimetinib, Figure S5: Induction of caspase 3/7 activity and apoptosis in MOLM-13 cells treated with idasanutlin and cobimetinib. Cell analysis by imaging cytometry of caspase 3/7 activity (**A**) and apoptosis (**B**) in MOLM-13 cells treated with the indicated amounts of idasanutlin and cobimetinib. Figure S6: Correlation of FLT3 and MDM2 protein levels in AML and normal leukocytes. MDM2 and FLT3 protein levels in cellular extracts of AML patient and normal leukocytes as determined by ELISA and normalized to GAPDH, Table S1: Genetic variants and drug responses in AML cell lines and patient samples.

## Abbreviations

AML	acute myeloid leukemia
FLT3	FMS like tyrosine kinase 3
ITD	internal tandem duplication
TP53	tumor suppressor p53
MDM2	mouse double minute 2 homolog
MEK	mitogen-activated protein kinase kinase
NPM1	nucleophosmin
RAS	rat sarcoma protein
GAPDH	glyceraldehyde 3-phosphate dehydrogenase
wt	wild type
mut	mutated
del	deleted
fs	frame shift
idasa	idasanutlin
cob	cobimetinib
NK	normal karyotype
