# Supplementary material for: The Cellular p53 Inhibitor MDM2 and the Growth Factor Receptor FLT3 as Biomarkers for Treatment Responses to the MDM2-Inhibitor Idasanutlin and the MEK1 Inhibitor Cobimetinib in Acute Myeloid Leukemia
Source: Cancers (Basel). 2018 May 31;10(6):170. doi: 10.3390/cancers10060170 (PMC6025168; doi:10.3390/cancers10060170)
Supplement: Supplementary file 1 [file cancers-10-00170-s001.pdf]

Supplementary Materials: The cellular p53 inhibitor MDM2 and the growth factor receptor FLT3 as biomarkers for treatment response to the MDM2-inhibitor idasanutlin and the MEK inhibitor cobimetinib in acute myeloid leukemia.

Katja Seipel, Miguel A. T. Marques, Corinne Sidler, Beatrice U. Mueller and Thomas Pabst

Supplemental Methods

Cytotoxicity Assays

AML cells were treated with the MDM inhibitor idasanutlin (RG-7388) (HY-15676, Medchemexpress, Monmouth Junction, NJ, USA) and the MEK inhibitor cobimetinib (GDC-0973, RG-7420) (S8041, Selleckchem, Lucerne, Switzerland). Cell viability was determined using the MTT-based *in vitro* toxicology assay (SIGMA-ALDRICH, St. Louis, MO, USA). For AML cell lines four independent assays (biological replicates) with four measurements (physical replicates) per dosage were collected and analyzed on GraphPad prism software. For cell lines data are depicted as XY graphs with mean and SD values and as dose-effect curves. For AML patient samples data are depicted as scatter plots with median values. Statistical analysis was done on GraphPad Prism in grouped analysis and significance calculated by Mann-Whitney test. Calculation of the combination index was done on CompuSyn software (version 1.0; ComboSyn, Inc. Paramus, NJ, USA). according to the method of Chou and Talalay [1] using the mean values of four biological replicates.

Measurement of mRNA Expression by qPCR

RNA was extracted from AML cells and quantified using qPCR. The RNA extraction kit was supplied by Macherey-Nagel, Düren, Germany. Reverse transcription was done with MMLV-RT (Promega, Madison, WI, USA). Real-time PCR was performed on the ABI7500 Real-Time PCR Instrument using FAST Start Universal probe master mix (Roche, Basel, Switzerland) and gene specific probes (Cat# 4331182, ThermoFischer Scientific, Waltham, MA, USA) Hs00180269_m1 (BAX), Hs01050896_ml (MCL1), Hs02758991_g1 (GAPDH) and Hs00355782_m1 (CDKN1A). Measurements for MCL1 and CDKN1A were normalized with GAPDH values (ddCt relative quantitation). Assays were performed in four biological replicates with four physical replicates each. Statistical analysis was done on GraphPad Prism software using two-tailed t-tests. Data are depicted in box graphs plotting median with Tukey range.

DNA Fragmentation Assay

AML cell lines were treated for 24 h with idasanutlin and cobimetinib. Genomic DNA was extracted using ZR-Duet DNA/RNA Miniprep kit (D7001, Zymo Research, Irvine, CA, USA.) and separated on 1% Agarose Gels. Intact genomic DNA was quantified by counting the pixels on the gel image and normalizing for DNA load per lane.

Flow Cytometry

For cell cycle analysis cells were fixed with 1% PFA (paraformaldehyde) in PBS for 30 min at 4 °C. Intracellular staining was done with Ki67-PE antibody (Cat# 12-5699-42, eBiosciences, ThermoFischer, Scientific, Waltham, MA, USA) in permeabilization buffer for 30 minutes at 4 °C followed by DAPI stain (Cat# 10 236 276 00, SIGMA-ALDRICH, St. Louis, MO, USA). Assays were performed in two biological replicates with two physical replicates each. Data analysis was done using FlowJo software (FlowJo LLC, Ashland, OR,USA).

Imaging Cytometry

Imaging cytometry was done on the NC-3000 cell analyzer (ChemoMetec, Allerod, Denmark) with reagents supplied by ChemoMetec. To determine induction of cell death apoptotic cells were stained with AnnexinV-CF488A conjugate (Biotium, Fremont, CA, USA) in AnnexinV buffer and Hoechst 33342 (10 ug/mL) for 15 min at 37 °C, followed by several washes. To determine caspase 3/7 activity cells were incubated with FAM-FLICA reagent (FAM-DEVD-FMK, Immunochemistry Technologies Bloomington, MN, USA) and Hoechst 33342 (10 ug/mL) for 60 min at 37 °C, followed by several washes. Propidium iodide was added shortly before imaging. For cell cycle analysis cells were incubated in lysis buffer with DAPI (10 ug/mL) for 5 min at 37 °C before imaging.

Measurement of Protein Levels by Western Blot

Total protein extracts were prepared by RIPA lysis. 100µg total protein extracts were separated on PAGE, transferred to nitrocellulose membrane and stained with mouse anti-p53 (sc-126, Santa Cruz Biotech, Dallas, TX, USA), rabbit anti acetylated p53 (K382) (Cell Signaling Technology #2525S, Leiden, The Netherlands), mouse anti-actin IgG (MAB 1501, Millipore, CA), mouse anti GADPH (G8796, SIGMA-ALDRICH, St. Louis, MO, USA) and rabbit anti phospho-eIF4E (Ser209) or rabbit anti-eIF4E (Cell Signaling Technology #9741 and #9742) followed by IRDye® 680LT goat anti-mouse IgG and IRDye® 800CW goat anti-rabbit IgG (LI-COR Biotechnology, BadHomburg, Germany). Membranes were scanned on LI-COR Odyssey Infrared Scanner (LI-COR Biotechnology, Germany). Bands were quantified on Odyssey application software.

Enzyme-Linked Immunosorbent Assay (ELISA)

It is very difficult to detect MDM2 and FLT3 in Western blots, due to high molecular weight of the proteins. We therefore used ELISA assays to quantify protein levels in AML patient samples and AML cell lines. Protein extraction was done according to standard protocol. In short, cell pellets were lysed in HEPES buffer containing 10 mM KCl, 1.5 mM $MgCl_2$, 1% NP40, 1 mM DTT and 10 mM PMSF. Supernatant containing cytoplasmic proteins was collected after centrifugation. The pelleted nuclei were lysed in HEPES buffer containing 420 mM NaCl, 1.5 mM $MgCl_2$, 25% glycerol, 0.2 mM EDTA and 1 mM PMSF. Nuclear and cytoplasmic protein extracts were pooled. Pooled cellular protein extracts were assayed in three physical replicates each. MDM2, FLT3 and GAPDH protein levels were determined with double-antibody Sandwich ELISA (SEG790Hu, SEB932Hu, SEA039Hu, Cloud-Clone Corp., Houston, TX, USA). MDM2 and FLT3 values were normalized with GAPDH values. Statistical analysis was performed using Mann-Whitney-test on GraphPad Prism software. Data are depicted as scatter plots with median values.

Supplemental Figures

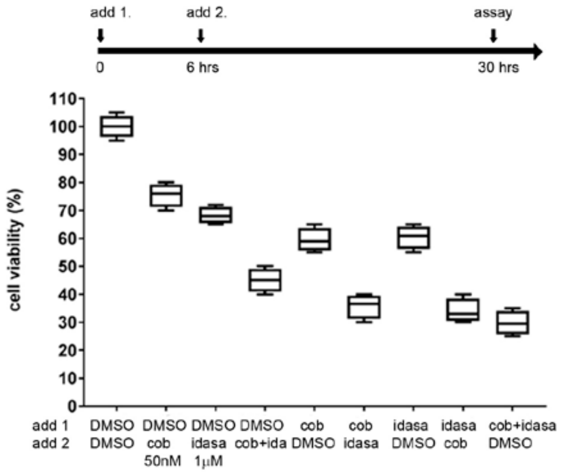

Figure S1. Synergistic effect on cell viability in OCI-AML2 cells treated with idasanutlin and cobimetinib independent of sequence of drug application. Cell viability measurements in cells pretreated for 6 h and treated for 24 h with idasanutlin and cobimetinib in all combinations. Idasanutlin pretreatment followed by cobimetinib treatment had the same effect on cell viability as cobimetinib pretreatment followed by idasanutlin treatment. Moreover, both sequential treatments had nearly the same effect on cell viability as 30 h of direct combination treatment.

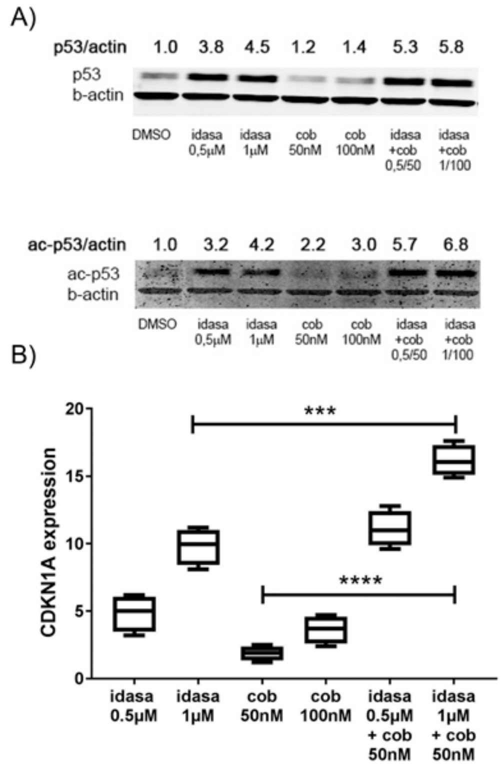

Figure S2. Induction of the tumor suppressor protein p53 and the cell cycle inhibitor CDKN1A gene in MV4-11 cells treated with idasanutlin and cobimetinib. Western blots for p53, acetylated p53 and b-actin (**A**) and quantitative PCR for *CDKN1A* mRNA normalized to *GAPDH* mRNA (**B**) in MV4-11 cells treated with the indicated amounts of idasanutlin and cobimetinib. Significance is denoted for $p < 0.0005$ (***); $p < 0.0001$ (****).

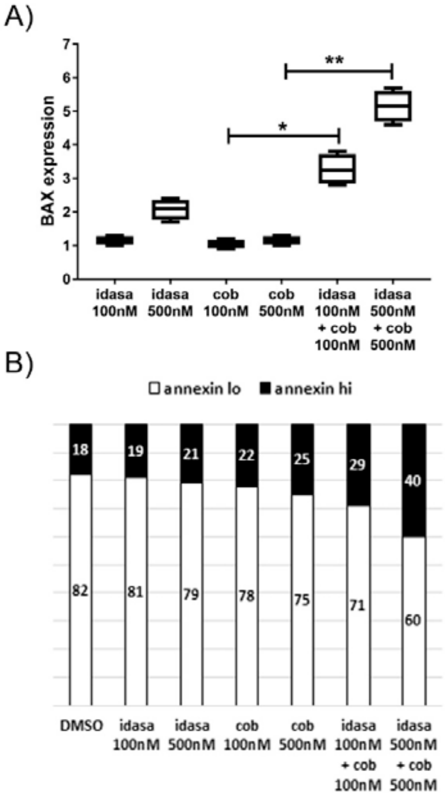

Figure S3. Induction of BAX gene expression and apoptosis in OCI-AML2 cells treated with idasanutlin and cobimetinib. Quantitative PCR for BAX mRNA normalized to GAPDH mRNA (**A**) and imaging cytometry results for annexin positive cells (**B**) in OCI.AML2 treated with the indicated amounts of idasanutlin and cobimetinib. Significance is denoted for $p < 0.05$ (*); $p < 0.005$.

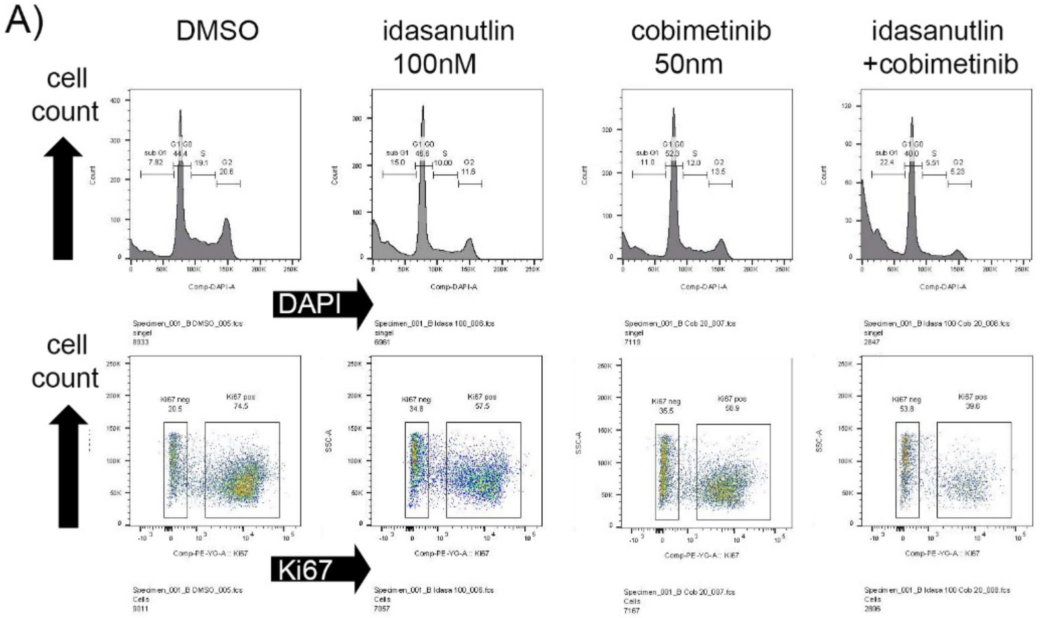

Figure S4. ***Cont.***

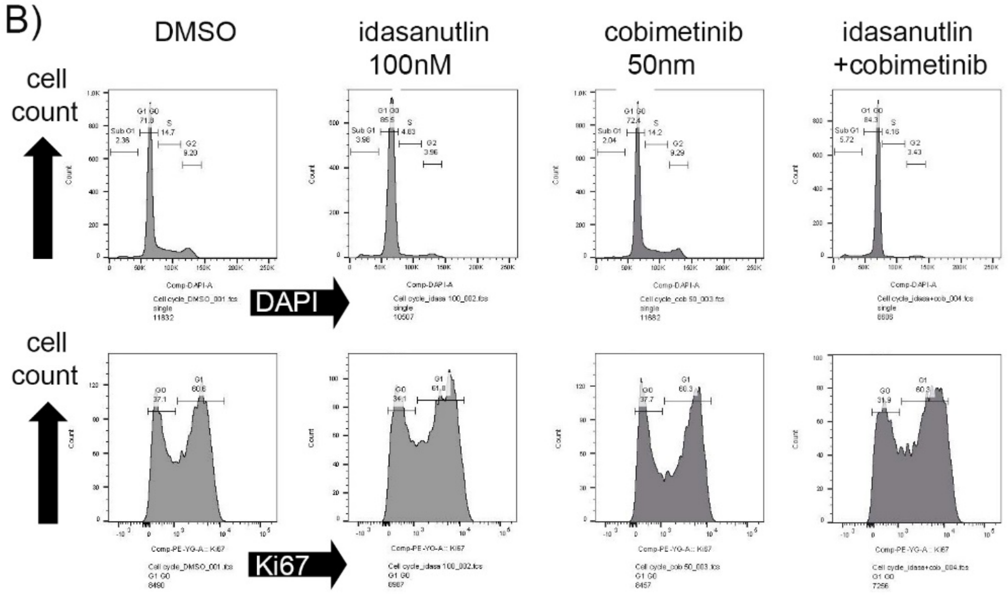

Figure S4. Induction of cell cycle exit and cell death in OCI-AML2 and MOLM-13 cells treated with idasanutlin and cobimetinib. Cell cycle analysis by flow cytometry measurements of DNA content (DAPI) and proliferation marker Ki-67 in OCI-AML2 (**A**) and MOLM-13 (**B**) treated with the indicated amounts of idasanutlin and cobimetinib.

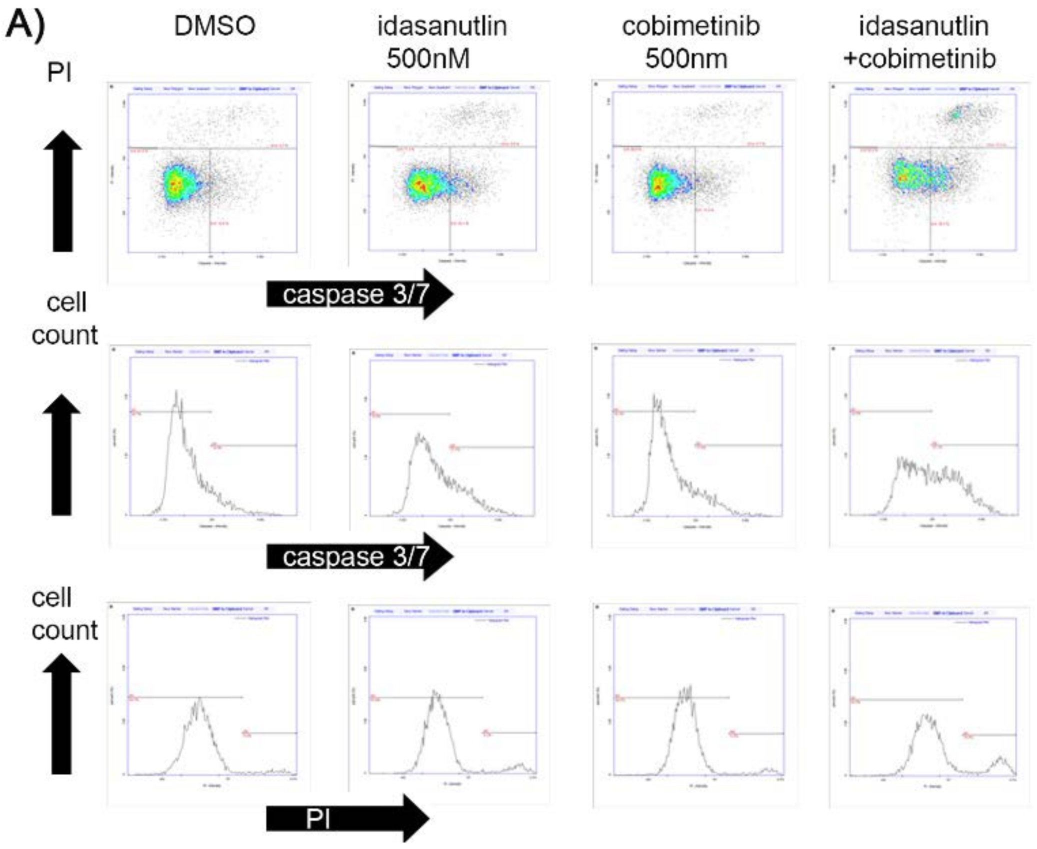

Figure S5. ***Cont.***

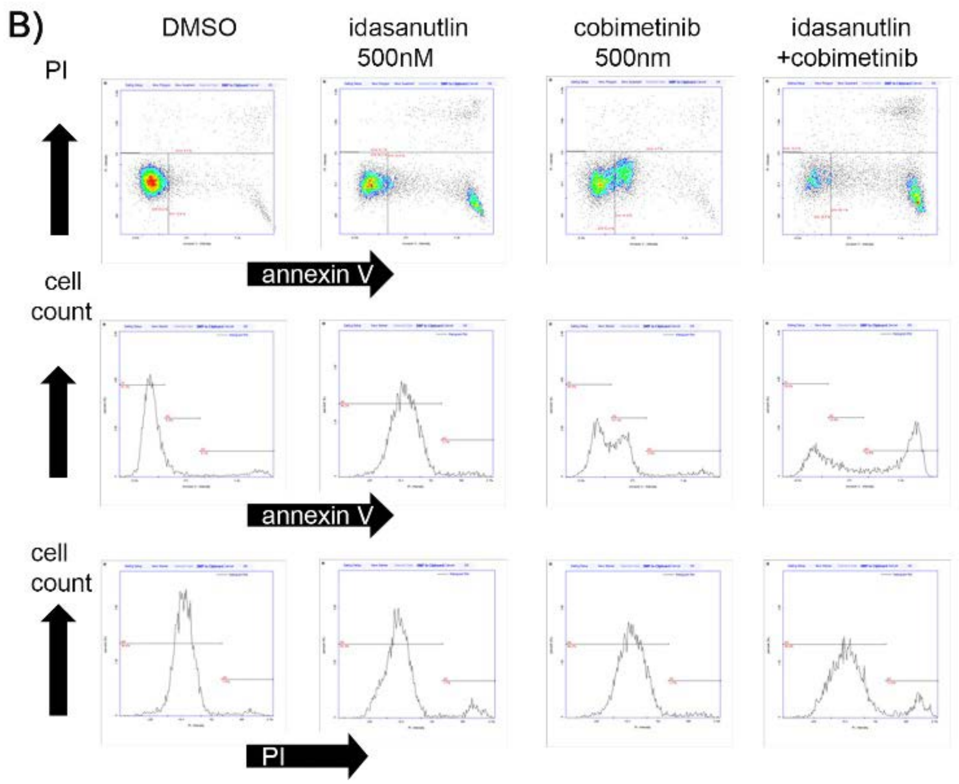

Figure S5. Induction of caspase 3/7 activity and apoptosis in MOLM-13 cells treated with idasanutlin and cobimetinib. Cell analysis by imaging cytometry of caspase 3/7 activity (**A**) and apoptosis (**B**) in MOLM-13 cells treated with the indicated amounts of idasanutlin and cobimetinib.

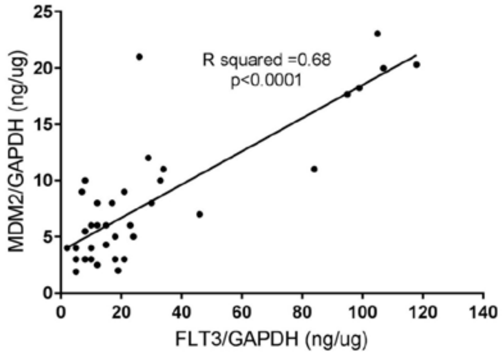

Figure S6. Correlation of FLT3 and MDM2 protein levels in AML and normal leukocytes. MDM2 and FLT3 protein levels in cellular extracts of AML patient and normal leukocytes as determined by ELISA and normalized to GAPDH.

Supplemental Table 1

Genetic variants and drug responses in AML cell lines and patient samples. (Separate file)

References

1. Chou, T.-C. Drug combination studies and their synergy quantification using the Chou-Talalay method. *Cancer Res.* **2010**, *70*, 440–446.

© 2018 by the authors. Licensee MDPI, Basel, Switzerland. This article is an open access article distributed under the terms and conditions of the Creative Commons Attribution (CC BY) license (http://creativecommons.org/licenses/by/4.0/).
